# Prospect of Stem Cell Conditioned Medium in Regenerative Medicine

**DOI:** 10.1155/2014/965849

**Published:** 2014-08-28

**Authors:** Jeanne Adiwinata Pawitan

**Affiliations:** Department of Histology, Faculty of Medicine, University of Indonesia, Jalan Salemba 6, Jakarta 10430, Indonesia

## Abstract

*Background.* Stem cell-derived conditioned medium has a promising prospect to be produced as pharmaceuticals for regenerative medicine. *Objective.* To investigate various methods to obtain stem cell-derived conditioned medium (CM) to get an insight into their prospect of application in various diseases. *Methods.* Systematic review using keywords “stem cell” and “conditioned medium” or “secretome” and “therapy.” Data concerning treated conditions/diseases, type of cell that was cultured, medium and supplements to culture the cells, culture condition, CM processing, growth factors and other secretions that were analyzed, method of application, and outcome were noted, grouped, tabulated, and analyzed. *Results.* Most of CM using studies showed good results. However, the various CM, even when they were derived from the same kind of cells, were produced by different condition, that is, from different passage, culture medium, and culture condition. The growth factor yields of the various types of cells were available in some studies, and the cell number that was needed to produce CM for one application could be computed. *Conclusion.* Various stem cell-derived conditioned media were tested on various diseases and mostly showed good results. However, standardized methods of production and validations of their use need to be conducted.

## 1. Introduction 

Data of the use of stem cells in various diseases are accumulating. Some studies reported beneficial effects of stem cell therapy in degenerative diseases such as myocardial infarction and revealed that stem cells cause tissue repair due to their ability to secrete trophic factors that exert beneficial impact on the damaged tissue, rather than their capacity to differentiate into the needed cells [1]. Various studies on stem cell-derived secreted factors showed that the secreted factor alone without the stem cell itself may cause tissue repair in various conditions that involved tissue/organ damage. The secreted factors are referred to as secretome, microvesicles, or exosome and can be found in the medium where the stem cells are cultured; thus, the medium is called conditioned medium (CM) [2].

The use of secretome containing CM has several advantages compared to the use of stem cells, as CM can be manufactured, freeze-dried, packaged, and transported more easily. Moreover, as it is devoid of cells; there is no need to match the donor and the recipient to avoid rejection problems. Therefore, stem cell-derived conditioned medium have a promising prospect to be produced as pharmaceuticals for regenerative medicine.

To date, no clinical trial that used CM for a certain disease has been reported, except two pilot studies on the use of adipose derived mesenchymal stem cell CM for hair follicle regeneration [3] and fractional carbon dioxide resurfacing wound healing [4] in human, which showed good results. The use of CM for therapy is very appealing and may be booming in the near future, as studies on the use of CM for various diseases are accumulating [1, 3–33]. Conditioned medium contains various growth factors and tissue regenerative agents, which were secreted by the stem cells. The fact that stem cells secrete various growth factors was also shown by various proteomic studies, which revealed the presence of various growth factors and other cytokines in the CM [5, 7–9, 13, 17, 20, 22, 28, 34, 35].

However, various studies reported the use of various kinds of stem cells and various methods to get the CM to cure various kinds of degenerative diseases in various animal models. Therefore, this systematic review aimed to investigate the various methods to get the CM and the various diseases that were treated, to get an insight into the various kinds of CM and their application benefit in various diseases.

## 2. Materials and Methods

We performed “all text” searches without time restriction on January 23, 2014, in Pubmed/Medline using keywords “stem cell” and “conditioned medium” or “secretome” and “therapy,” “all text” searches in Cochrane library (trials) using keywords “secretome” or “conditioned medium,” and “all text” searches in ClinicalTrials.gov using keywords “stem cell” and “conditioned medium” or “secretome” and “therapy.” In addition, relevant existing articles in our library were added.

Inclusion criteria are all studies that used CM for a certain disease. Exclusion criteria are studies that did not contain complete data concerning subject condition/disease model, source of CM, and outcome of treatment with CM.

Data collection is as follows: treated conditions/diseases, type of cell that was cultured, detailed composition of medium and supplements that was used to culture the cells, culture condition (hypoxia or normoxia) to get the CM, CM processing, growth factors, and other secretions that were analyzed; method (mode) of application and outcome of CM application were noted, grouped, and tabulated.

Data synthesis is as follows: data were grouped according to treated disease and cell types that were used to produce the CM. Further, to know the growth factor yields of the various types of cells, when available, growth factor levels were tabulated and grouped according to types of cells that yielded the growth factor containing conditioned medium, in relation to the number of cells, type and duration of culture, and processing of the conditioned medium. When the data was available, the number of cells that were needed to produce the CM for one application was computed.

## 3. Results and Discussion

We got 39 articles that met the inclusion criteria, and 7 were excluded due to incomplete data. Various conditions/diseases were treated by various cell-derived CM and mostly showed promising results (Table 1).

The various conditioned media, even when they were derived from same kind of cells, were produced by different condition, that is, from different passage, number of cells, culture medium, and culture condition (Table 2). The growth factor yields of the various types of cells can be seen in Table 3, and the cell number that is needed to produce CM for one application can be seen in Table 4.

Various studies showed that conditioned medium have been tested in various kinds of diseases/conditions (Table 1) [1, 3–33], that is, alopecia [3, 5], acute and chronic hind limb ischemia [6–9], acute and chronic wound healing [4, 10–14], myocardial infarct [1, 15–18], acute liver injury/failure [19–22], cerebral injury/ischemia/stroke [23–27], spinal cord injury [28], lung injury [32], and bone defect [33], and showed improvement of the conditions. Moreover, chronic kidney disease that was treated using human embryonic stem cell-derived mesenchymal stem cell (huESC-MSC) CM showed decreased systolic blood pressure and proteinuria and improvement in tubular and glomerular damage, renal blood flow, and glomerular filtration rate [29]. However, nephropathy that was treated using CM from human umbilical cord blood unrestricted somatic stem cell (huUCB-USSC) or mouse bone marrow mesenchymal stem cell (mBM-MSC) CM did not show improvement in serum urea and creatinine level, histopathological damage, and physical activity score [30]. Moreover, prevention of cancer using human mesenchymal stem cell line CM showed increased tumor cell proliferation and vascularization [31].

In the two cases of kidney disease, it can be concluded that CM from hu-ESC-MSC can improve the condition, and the needed growth factor level is presumably enough as CM processing includes a 25-time concentration step [29]. However, for hu-UCB-USSC or mBM-MSC-CM, lack of data concerning CM processing and growth factor level of the CM [30] prevent further analysis to conclude whether the failure to improve the condition is due to the lack of certain growth factor or due to the level of growth factors that was too low to give an effect.

The conditioned medium can be harvested from various kinds of cells (Table 2). Moreover, there are various methods to get the conditioned medium, which may interfere with the growth factor types and levels that were harvested by the methods. Only some of the various studies using CM checked the growth factor levels (Table 3) [5–10, 13, 17, 20, 22, 24, 25, 27, 28, 33, 36] and the same type of cells yielded different growth factor levels, when cell number, culture medium and condition, and CM processing were different [6, 24]. Moreover, growth factor measures also differed, that is, pg/mL or ng/mL [6, 8, 10, 13, 17, 24, 27, 28, 33], pg/*μ*g DNA [9, 36], fg/cell [25], spot density [5], and positive/negative [20] (Table 3). The measure of pg/*μ*g DNA and fg/cell can be computed into pg or ng/mL provided the DNA content/cell and cell number is known. However, in some studies, the exact cell number that was used to produce the CM was not mentioned [7, 8, 10, 13, 28, 33, 36]. In addition, most studies measured different sets of growth factors and other cytokines/factors (Table 3).

### 3.1. Culture Medium and Supplement

Some studies used fetal bovine serum or other supplement containing complete medium, while other studies used serum-free media. Moreover, the basal media used were variable, for example, *α*MEM, DMEM, DMEM/F12, M199, EBM2, EGM-2, in vivo 15, or chemically defined medium, and the same type of cell might be cultured in different kind of basal medium (Table 2). Culture medium in* in vitro* culture represents microenvironment in* in vivo* condition and may determine cell fate and thus cell secretion [37]. Therefore, the same type of cells may secrete different level of growth factors when they were cultured in different medium, as can be seen in Table 3 [25, 27].

### 3.2. Culture Duration

Production of CM varies in culture duration from sixteen hours to five days (Table 3). In case complete medium was used, short culture duration might leave certain serum derived growth factors that was not consumed by the cells and might add to the growth factor level, or, on the contrary, suppress growth factor secretion by the cells. Possibility of the presence of residual growth factor from the medium can be seen in a study, which showed that medium without cell contained a TGF-b1 level of 2.49 ± 2.39 pg/mL (Table 3) [24].

### 3.3. Culture Condition

Some studies produce CM from cell culture in normoxia (O_2_ level 20-21%) and variable oxygen deprived (hypoxia O_2_ level 0.5%, 1%, 1.5%, and 2%) condition (Table 2). Some studies on various stem cells showed that most growth factors were upregulated in hypoxia condition, for example, vascular endothelial derived growth factor (VEGF) [5, 8, 27], hepatocyte growth factor (HGF) [8, 27], platelet derived growth factor (PDGF) [5, 8], placenta growth factor (PlGF) [25], and insulin-like growth factor II (IGF-II) [5], except epidermal growth factor (EGF) that was downregulated [5]. However, another study showed the contrary, that is, down regulation of VEGF and HGF in hypoxia condition [25].

Most studies produced CM in monolayer culture, but several studies used spheroid cultures (Table 3). Spheroid cultures need a special handling and equipment (spinner flask) but yield more cells compared to conventional monolayer cultures, and thus more secreted factors [6, 24] (Table 4). In addition, cells located at the center of the spheroid may be in relative hypoxic condition compared to cells on the surface, thus further increasing certain growth factor yield.

### 3.4. Secreted Factor's Role in Improvement of Diseases

Various cytokines were secreted by stem cells into the CM, and they played a role in the improvement of various diseases/conditions. Those cytokines can be grouped into growth factors, proinflammatory and anti-inflammatory cytokines, and other cytokines. Various studies used various methods to assess various cytokines in the conditioned CM, from the conventional ELISA assays [6, 10, 24, 25, 27, 33, 36] to proteomic profiling methods [5, 7–9, 13, 17, 20, 22, 28, 34, 35].

#### 3.4.1. Growth Factors

Growth factors that are secreted by various kinds of stem cells are vascular endothelial derived growth factor (VEGF) [5, 6, 8, 10, 13, 17, 24, 25, 27, 33, 36], platelet derived growth factor (PDGF) [5, 8, 10, 13, 24], epidermal growth factor (EGF) [5, 10, 13, 20], insulin-like growth factor I (IGF-I) [33, 36], insulin-like growth factor II (IGF-II) [5], hepatocyte growth factor (HGF) [6, 8, 20, 25, 27, 33], fibroblast growth factor 2/basic fibroblast growth factor (FGF-2/bFGF) [6, 7, 10, 13], keratinocyte growth factor/fibroblast growth factor 7 (KGF/FGF-7) [10, 20], platelet derived endothelial cell growth factor (PDEGF) [20], heparin binding epidermal growth factor (HEGF) [20], placenta growth factor (PlGF) [25], neural growth factor (NGF) [28], and brain derived neurotrophic factor (BDNF) [28].

Further, studies that analyzed various growth factors reported the presence of the various growth factors, which were secreted by various stem cells into their conditioned medium (Table 3), except for human MSC (Lonza) that did not secrete FGF-2, PDGFBB, BMP-2, and SDF-1 but secreted IGF-1, VEGF, TGF *β*1, and HGF [33]. Moreover, different culture condition and medium may yield different level of growth factor secretions [6].

#### 3.4.2. Pro- and Anti-Inflammatory Cytokines

Anti-inflammatory cytokines that are secreted by stem cells are TGF*β*1 [9, 10, 20, 24, 33] and some interleukins (IL), that is, IL-6 [9, 13, 17, 25, 28], IL-10, IL-27, IL-17E, IL-13, IL-12p70, and IL-1 receptor antagonist (IL-1ra) [20], while the secreted proinflammatory cytokines are IL-8/CXCL-8 [8, 9, 13], IL-9 [13, 35], and IL-1b [20].

#### 3.4.3. Other Cytokines

Other secreted factors are leptin [7], angiogenin [8], granulocyte colony stimulating factor (GCSF) [5, 10], granulocyte macrophage CSF (GM-CSF) [5, 10, 13], macrophage CSF (MCSF) [5], fractalkine [13], monocyte chemotactic protein (MCP-1) [9, 13, 17, 20], serpin E-1 [20], endostatin/collegen XVIII [20], UPA, thrombospondins 1 and 2 [20], tissue inhibitor of metalloproteinase-1 (TIMP-1) [20], IGF binding protein (IGFBP) [5, 20], stem cell-derived factor 1 (SDF-1)/CXCL-12 [6–9, 20, 25], adrenomedullin (ADM) [25], Dickkopf-1 (DKK-1) [25], and receptors, that is, MCSF receptor (MCSFR) [5] and PDGF receptor (PDGFR) [5].

### 3.5. Translation of Conditioned Medium Usage in Patients

In conditioned medium, various factors may be present as a cocktail and act in concert to promote regeneration. Therefore, it is important to analyze a complete set of growth factor and cytokine levels for every kind of stem cell-derived conditioned medium and to know the culture condition, conditioned medium processing, and diseases/conditions that are responsive to a certain conditioned medium treatment. When the content of the various cytokines in a certain conditioned medium is known, the result of the conditioned medium on a certain disease/condition can be determined, and the way to translation into patients is open.

From studies that analyzed VEGF level we can conclude that most stem cells secrete VEGF. As VEGF plays a role on angiogenesis [36] that is important in regeneration of injured/damaged tissues/organs, various stem cell-derived conditioned media are able to cure various diseases and will have more impact on diseases with ischemia. In addition, VEGF may prevent apoptosis in hypoxic condition, thus preventing further damage [6].

Concerning angiogenesis, other than VEGF, other growth factors that may play a role in angiogenesis are FGF2 [7, 38], EGF [7], HGF [7, 8], PlGF [7], SDF-1 [7], PDGF [7, 38], TGF*β*1 [38], and PDEGF [39]. In addition, various cytokines, that is, interleukin [38], IL-8 [8, 9, 13], chemokines [38], monocyte chemotactic protein (MCP-1) [9, 13, 17, 20], leptin [7], angiogenin [8], and endostatin/collagen XVIII [20], also play a role in angiogenesis.

Moreover, FGF2 is a more potent angiogenic factor compared to VEGF, with additional effect on proliferation of fibroblasts, preadipocytes, and endothelial, epithelial, and neural stem cells, on migration of neural crest derived glial and myogenic cells and on differentiation of neuroepithelial cells into mature neurons and glial cells [38].

Other growth factors contribute in the regeneration of injured/damaged tissue organs, with special emphasis on proliferation, that is, PDGF for connective tissue, glial, and other cells, EGF for mesenchymal, glial, and epithelial cells, and IGF-I and IGF-II for various kinds of cells [40]. In addition, PlGF that is a member of VEGF family increases the activity of VEGF* in vitro* and* in vivo* [41], KGF inhibits oxidative stress induced epithelial cell death [42], NGF promotes neurite outgrowth and neural cell survival [40], BDNF is neuroprotective, promotes cell survival, and reduces astroglial scar formation [28], and some growth factors, including HEGF, FGF-7, EGF, and HGF promote liver regeneration [20].

Proinflammatory cytokines that play a role in regeneration are IL-1b due to its liver protective role [20], IL-8 due to its angiogenic activity [8, 9, 13], and IL-9 due to wound healing promotion activity [13, 43]. In addition, anti-inflammatory cytokines prevent inflammation and promote liver regeneration [20].

Other cytokines, that is, UPA and thrombospondins 1 and 2, promote liver regeneration [20], serpin E-1 [20] and SDF-1 [6–9, 20, 25] promote tissue repair [20], TIMP-1 and IGFBP [5, 20] prevent apoptosis [20], ADM causes vasodilatation and reduces cellular oxidative stress and apoptosis [25], DKK-1 initiates bone marrow stem cell proliferation [25], and fractalkine prevents apoptosis [13, 44].

Various colony stimulating factors, that is, granulocyte colony stimulating factor (GCSF) [5, 10], granulocyte macrophage CSF (GM-CSF) [5, 10, 13], and macrophage CSF (MCSF) [5], may recruit various resident stem cells/progenitor cells including endothelial progenitors to site of injury/damage and promote wound healing process [10, 13] or hair growth [5].

MCSF receptor (MCSFR) [5] promotes myeloid progenitor, mononuclear phagocyte, and placental trophoblast growth and development [45], and PDGFR [5] may interact with various signaling molecules or integrin to cause cell proliferation, motility, differentiation, or survival by apoptosis inhibition [46].

Moreover, one factor may contribute to more than one mode of regenerative action, such as MCP-1 that is involved in angiogenesis [9, 13, 17, 20] and liver protection activity [20]. Further, for production of CM to be applied in various human diseases, data from animal studies that showed promising outcome are very valuable.

#### 3.5.1. Production of CM for Translation into Various Human Diseases

**Figure 1:**
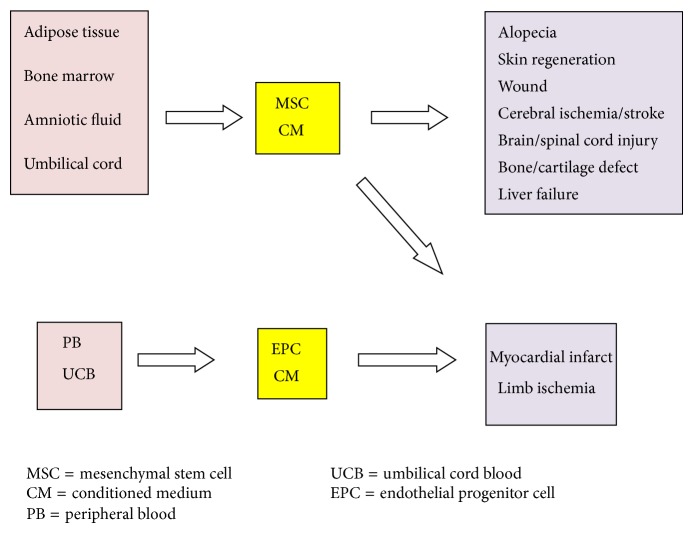
Various possible applications of CM for various conditions.

To use CM for various human diseases, production method of the CM needs to be standardized in terms of the type and number of cells that were needed to produce the CM, culture medium and condition, and conditioned medium processing. In addition, the volume and mode of delivery are also important. As various studies used various numbers and type of cells and various doses of CM, it is important to know the number of cells that yielded the CM for one application, which may be interpolated for human studies. Therefore, in Table 4 we summarized all data that may be needed for interpolation into human studies, that is, diseases that were treated, species and age or body weight of the animal, type of cell, culture medium and condition, number of cells to produce CM for one application, volume, and mode of application. Moreover, various possible applications of CM for various conditions are summarized in Figure 1.

In addition, for translation into patients, it is very important to analyze and to note the various cytokine contents of the various conditioned media. Further, for every conditioned medium with known cytokine content, validation of its use on various diseases needs to be conducted. Finally, the possibility of promotion of existing cancer should be tested for every CM, and caution should be taken before CM therapy to ensure that the recipient is free from cancer.

Advantages of production of various CM for patients lie in the possibility of mass production by pharmaceutical companies, when production methods have been standardized. Conditioned media are not like stem cells that need a good manufacturing practice (GMP) facility to be applied to patients [47]. When CM has been packaged properly, it can be transported easily as drugs and does not need cryopreservation, such as that the stem cells need. However, compared to stem cells that may survive for a rather long period, CM needs to be given more frequently, as cytokines' and growth factors' half-lives are mostly shorter [48, 49], which is a disadvantage for the patients but will give more profit to pharmaceutical companies.

## 4. Conclusion

Various stem cell-derived conditioned media were produced by various methods and processing and tested on various diseases and mostly showed good results. However, standardized methods for various conditioned media production and validations of their use on various diseases need to be conducted.

## Figures and Tables

**Table 1:** Studies on various subjects, conditions, source of conditioned medium, and outcome.

Condition/disease	Subject	Source of conditioned medium	Outcome	Reference number
Alopecia—ID	Human	Hu-AD-MSC	Increased hair growth	[3]

Bald—SC	C_3_H/HeN nude mice	Hu-AD-SC	Hair growth	[5]

Acute hind limb ischemia—direct IM	Female athymic mouse	Hu-AD-SC	Decreased LL and F Increased BF, angiogenesis, endothelial growth, homing, and AA	[6]
	SCID mice	Hu-ESC—endothelial cells	Vascularization and BF: CM restored defective diabetic PB derived PAC	[7]

Chronic hind limb ischemia—7–10 days IM	Male nude athymic	Hu-PB-MNC-EPC Hu-UC-HUVEC	Increased hind limb BF	[8]
	Male NOD-SCID mouse	Hu-AF—SC—Ckit (+)	Increased arteriogenesis, capillary density, total perfusion area, and mobility, and decreased muscular deg	[9]

Skin wound direct—ID, [11] SC [10, 12]/topical application [4, 13]	Human	Hu-AD-SC	Enhanced wound healing Reduced adverse effects	[4]
	BALBc nude mice	(i) Hu-UCB-MNC ⟶ UCB-SC (endothelial + MSC) (ii) HUVEC	Faster wound healing: UCB-SC was better than HUVEC	[10]
	Diabetic immunodeficient mice	Hu-UCB-CD34-EPC	Faster wound closure Less granulation tissue area More neovascularization	[11]
	Male db/db (diabetic) mice	Hu-UC-MSC	Faster wound closure Increased capillary density	[12]
	BALBc-nude mouse	(i) Hu-ESC—derived EPC (ii) Hu-UCB-EPC	Faster wound healing, granulation, and reepithelization: huESC-EPC was better than UCB-EPC	[13]

Skin wound—48 hour after wound—SC	Male NOD-SCID mice	Hu-BM-MSC	Faster wound healing	[14]

MCI—direct—peri-infarct injection	Male SCID or C57BL/6 mouse	Hu-AD-SC	Improved cardiac function Reduced infarct size Effect of huAD-SC > CM	[1]

MCI—end of 2nd hour R—IC	Female L pig	Porcine PB-EPC	Reduced IZ-A and infarct size Increased IZ angiogenesis IZ cardiomyocyte hypertrophy Improved LV contractility and relaxation	[15]

MCI—4 hours—IV (jugular vein)	DL pig	Hu-ESC-MSC	Increased capillary density Reduced infarct size Preserved S-D performance	[16]

MCI—48 hours-IM yo	Rat nude athymic	Hu-BM-derived MPC	Improved LV function Reduced LV dilation, myocyte A, and fibrosis Increased neovascularization	[17]

MCI—5 min before R—IV, -at R—IC	Female DL pig	Hu-ESC derived MSC	(i) Reduced infarct size and A (ii) Improved S-D performance	[18]

MCI—5 min before R—IV—(tail)	Mouse	Hu-ESC derived MSC	Reduced infarct size (>1000 kD/100–220 nm) = 10–220 nm < 10–100 nm	

RSLT—direct—IV—(penile)	Male SD rat	Rat BM-MSC	Reduced LIB and PIC Increased survival	[19]

Acute hepatic failure—24 hours—intrahepatic (left liver lobe)	CCl4 injured SCID/NOD mice	1-Hu-AF MSC 2-AF-MSC-hepatic progenitor-like cells (HPL)	(i) AST, ALT decreased (ii) Liver phenotype improvement HPL was better than MSC-CM	[20]

Fulminant hepatic failure—24 hours—IV (penile)	Male SD rat	Hu-MSC	Reduced ALT, AST, TNF*α*, IL6, and IL1-rec-A level, and HP, ICI, and A Increased IL10 level, liver regeneration, and survival	[21]
	Male SD rat	Hu-BM-MSC	Reduced panlobular leucocyte infiltrate, hepatocellular death, and bile duct duplication and increased survival	[22]

Focal cerebral ischemia—72 hours—intranasal	Male SD rat	(i) Hu-SC-EDT (ii) BM-MSC (Lonza)	Increased migration-diff—endogenous NPC, vasculogenesis, and motor function, and reduced infarct size (Hu SC-EDT = BM-MSC)	[23]

Ischemic stroke—after 8 days—lateral ventricle infusion	Male SD mice	Hu-AD-MSC	Motor function maintained, reduced infarct volume, neural cell A, and astrogliosis, and increased microvessel	[24]

Cerebral ischemia infarction—1 day—IC/intracardiac (LV) injection	immunodeficient mice	(i) Hu-BM-MSC (ii) Hu-BM-CD133 (iii) Hu-BM-p75 (iv) Hu-fibro	Reduced cortical infarct volume (huBM-CD133-CM < huBM-MSC-CM < hufibroCM < huBM-p75CM)	[25]

Fluid percussion-TBI—direct IV jugular vein	Male SD rat	Hu-BM-MSC	Reduced neuron loss, A, neuron A, infarction volume, and motor deficit Increased VEGF(+) cells	[26]

Fluid percussion TBI—12 hours after—IV	Male SD rat	Hu-BM-MSC	Decreased brain damage volume, brain damage incidence, and neuron A (hypoxia < normoxia) Increased motor/cognitive function and neurogenesis (hypoxia > normoxia)	[27]

Contusion spinal cord injury—direct	Female Wistar rat	Rat-BM-MSC	Increased motor recovery	[28]

Chronic kidney disease—week 5—IV (tail)	Male Le rat	Hu embryonic MSC—stable—80 population doublings	Decreased systolic BP, proteinuria, and tubular + glomerular damage Increased inulin and PAH clearance, glomerular endothelium, and DNA repair	[29]

Nephropathy—24 hours—IV (tail)	Mouse BALBc	(i) Hu-UCB-USSC (ii) Mouse BM-MSC	No improvement in serum urea and creatinine, HP, and physical activity score	[30]

Normal—cancer cell line + CM xenograft	BALB mice	Hu-MSC (cell line)	Increased tumor cell proliferation (PCNA) and vascularization	[31]

VILI—before induction—IV—(tail)	Male C57BL/6 mouse	Mouse-iPSC	Reduced tidal volume, and bronchial microstructure restored	[32]

Intrabony periodontal defect direct—implant	Hybrid dog	Hu-MSC (Lonza)	Increased alveolar bone and cementum regeneration	[33]

ID: intradermal, IM: intramuscular, SC: subcutaneous, MCI: myocardial infarct, R: reperfusion, IC: intracoronary artery, IV: intravenous, Imyo: intramyocardial, LV: left ventricular, RSLT: 50% reduced size liver transplantation, TBI: traumatic brain injury, VILI: ventilator induced lung injury, SCID: severe combined immunodeficient, NOD: nonobese diabetic, SD: Sprague-Dawley, DL: Dalland Landrace, L: Landrace, W: Wistar, Le: Lewis, hu: human, AD: adipose tissue derived, MSC: mesenchymal stem cells, SC: stem cell, ESC: embryonic stem cell, PB: peripheral blood, MNC: mononuclear cell, UC: umbilical cord, UCB: UC blood, BM: bone marrow, EPC: endothelial progenitor cell, HUVEC: human umbilical vein endothelial cell, AF: amniotic fluid, EDT: exfoliated deciduous tooth, MPC: mesenchymal progenitor cell, USSC: unrestricted somatic stem cell, iPSC: induced pluripotent stem cell, LL: limb lost, F: fibrosis, BF: blood flow, AA: antiapoptosis, CM: conditioned medium, PAC: proangiogenic cells, deg: degeneration, IZ: infarct zone, A: apoptosis, ALT: alanine amino transferase, AST: aspartate aminotransferase, HP: histopathology, ICI: immune cell infiltration, S-D: systolic-diastolic, LIB: liver injury biomarker, PIC: proinflammatory cytokine, Hu-SC-, IL1-rec-A: IL1 receptor antagonist, NPC: neural progenitor cell, PAH: para amino hippuric acid.

**Table 2:** Cell type, medium, culture condition, cell number, duration, passage, and processing of conditioned medium.

Reference number	Cell type	Medium/vessel	Culture condition	Cell number	Duration	Passage	CM processing
[7]	Hu-ESC-EC HUVEC	EBM2	NA	NA	5 days	NA	NA
[13]	(i) Hu-ESC-CD133/KDR-EPC (ii) Hu-UCB-EPC	EGM-2 (Lonza)—15 mL 150 mm culture dish	NA	80%	48 hours	P5–8	Conc. 50x 10 kD
[16] [29]	Hu-ESC-MSC Hu-ESC-MSC	DMEM—insulin, transferrin, selenoprotein, FGF2, PDGF-AB, glutamine, and *β*-ME	NA NA	NA NA	3 days 3 days	≥80 PD 80 PD	Conc. 25x 10 kD—220 nm → 0.5 mg/mL protein
[18]	Hu-ESC-MSC	Chemically defined medium	NA	NA	3 days	80 PD	Conc. 25x 10 kD^a^
[9]	Hu-AF SC—Ckit (+)	*α*MEM Six-well plate	5% CO_2_	150.000 70%	16 hours	NA	from 500.000 cells—1 mL—Conc. → 80 *µ*L
[20]	1-Hu-AF-MSC 2-hu-AF-MSC-HPL	DMEM 0.5% FBS 25 cm^2^ TC flask	5% CO_2_	1.5 × 10^6^ 80%	24 hours	P5–13	Conc. 25x 3 kD
[8]	(i) Hu-PB-MNC-EPC (ii) HUVEC	EBM2 (Lonza) 1% FBS	1.5% O_2_, 5% CO	NA	72 hours	NA	NA
[15]	Porcine PB-MNC-EPC	Ex vivo 15 (Lonza)—VEGF 1 ng/mL—FC plate	5% CO_2_	From 30–40 mL PB	48 hours	P0	Centr. 600 g 5 min, 0.2 *μ*m—ice
[12]	Hu-UC-MSC	M199	5% CO_2_	NA	24 hours	P3	Conc.- 0.2 *μ*m
[10]	(i) Hu-UCB-MNC-SC (endothelial + MSC) (ii) HUVEC	EGM-2–15 mL 150 mm culture dish	5% CO_2_	80%	48 hours	P5–8	Conc. 50x—10 kD
[11]	Hu-UCB-CD34-EPC	M199 basal medium	5% CO_2_	1 × 10^6^	24 hours	NA	Conc.
[30]	(i) Hu-UCB-USSC (ii) mBM-MSC	Ultra CULTURE medium 7.5% BABLc serum	5% CO_2_	60%	48 hours	NA	NA
[14]	Hu-BM-MSC	*α*MEM 10% FBS	5% CO_2_	2 × 10^7^/flask 60–70%	Till 60–70%	P5	Conc. 50x—5 kD 5 flask → 100 *μ*L
[22]	Hu-BM-MSC	NA-0.05% BSA	NA	2 × 10^6^	24 hours	P3–7	Conc. 25x—3 kD
[26]	Hu-BM-MSC	DMEM-0.05% BSA	normoxia	2 × 10^6^	24 hours	P3–7	Conc. 25x—3 kD
[27]	Hu-BM-MSC	DMEM-0.05% BSA	5% CO_2_ 0.5% O_2_	2 × 10^6^ → split 1 : 2 → confl	24 hours	P3–7	Conc. 25x 3 kD
[17]	Hu-BM-MNC-stro-3-MPC	*α*MEM	NA	1 × 10^6^ MPC	NA	P5	Conc.
[25]	(i) Hu-BM-MSC (ii) Hu-BM-CD133 (iii) Hu-BM-p75 (iv) Hu-fibro	*α*MEM	5% CO_2_ (i) 1% O_2_ (ii) 21% O_2_	1 × 10^6^ 90%	48 hours	P4-5	0.2 *µ*m—80°C conc. 40x—5 kD
[28]	Rat-BM-MSC	DMEM T75 flask	NA	90%	48 hours	P2–4	Conc. 40x—10 kD: 10mL → 250 *µ*L 0.22 *µ*m—80°C
[19]	Rat BM-MSC	DMEM-0.05% BSA 10 mL	NA	80–90%	12 hours	P3-4	Conc. 25x 3 kD
[23]	(i) Hu-SC-EDT (ii) BM-MSC (Lonza)	DMEM	5% CO_2_	4 × 10^5^	48 hours	P3–5	3000 rpm—3 min → supernatant
[3]	Hu-AD-MSC	NA	Hypoxia	NA	NA	NA	Conc.-freeze dried
[24]	Hu-AD-MSC	*α*MEM 70 mL	Spheroid 1% O_2_—5% CO_2_	4.2 × 107	2 days	NA	Centr.
[6]	Hu-AD-SC	CRM/*α*MEM CRM—hu allo 10% *α*MEM—FBS 10%	1% O_2_—5% CO_2_	2.5–3 × 10^5^/mL—24 mL—150 cm dish	2 days	Up to P5	Centr.
		CRM/*α*MEM CRM—hu allo 10% *α*MEM FBS 10%	Spheroid 1% O_2_—5% CO_2_	6–12 × 10^5^/mL—30 *μ*L → 70 mL flask			
[1]	Hu-AD-SC	NA	NA Reference [13, 14]	1 × 10^5^ →80%	24 hours	NA	Centr. 300 g 5 min—220 nm
[5]	Hu-AD-SC	DMEM/F12	2% O_2_ 5% CO_2_	4 × 10^5^	72 hours	P4-5	Centr. 300 g 5' 0.22 *µ*m, 3 kD
[23]	Hu-MSC	DMEM-0.05% BSA 15 mL—175 cm^2^ flask	NA	1 × 10^6^ 70–80%	24 hours	NA	Conc. 25x 3 kD
[31]	Hu-MSC (cell line)	DMEM-10% FBS 5 mL	5% CO_2_	70%	48 hours	NA	100,000 g—1 hour → supernatant 0.22 *µ*m
[33]	Hu-MSC (Lonza)	DMEM	5% CO_2_	70%	48 hours	P3–9	4°C or −80°C
[32]	Mouse-iPSC	NA	NA	NA	NA	NA	NA

Hu: human, ESC: embryonic stem cell, EC: endothelial cell, HUVEC: human umbilical vein endothelial cell, EPC: endothelial progenitor cell, AF: amniotic fluid, SC: stem cell, MSC: mesenchymal SC, HPL: hepatic progenitor-like cell, AD: adipose tissue derived, PB: peripheral blood, MNC: mononuclear cell, UC: umbilical cord, UCB: UC blood, m: mouse, BM: bone marrow, MPC: mesenchymal progenitor cell, fibro: fibroblast, EDT: exfoliated deciduous tooth, iPSC: induced pluripotent SC, TC: tissue culture, NA: not available, FBS: fetal bovine serum, allo: allogenic serum, BSA: bovine serum albumin. ∗Filtered 220 nm → 10 nm → 100 nm yielded 10–220 nm versus 10–100 nm (<1000 kD) versus 100–220 nm (>1000 kD). FC: fibronectin coated, P: passage, PD: population doubling, CM: conditioned medium, conc.: concentrated, centr.: centrifugation.

**Table 3:** Growth factor level from various cell sources, culture duration, cell number and processing of conditioned medium.

Reference number	Cell source	Culture/duration	Cell number/processing	Growth factor level	
[7]	Hu-ESC-EC	Monolayer —5 days	NA	Angiogenic cytokine: VEGF, SDF-1, PlGF, leptin, EGF, bFGF, and HGF: CM DM-PAC < CM cPAC Angiogenic cytokine: VEGF, PDGF, ICAM-1, EGF, and bFGF: CM ESC-EC > CM c-PAC = CM D-PAC	

[13]	(i) Hu-ESC-CD133/KDR-EPC (ii) Hu-UCB-EPC	Monolayer—48 hours	80% in 150 mm culture dish/Conc. 50x	EGF	ESC-EPC versus CB-EPC versus EGM-2 12584 versus 12654 versus 9 pg/mL
				FGF-2	383 versus 652 versus 61
				Fractalkine	1605 versus 150 versus 133
				GM-CSF	755 versus 323 versus 313
				IL-6	4332 versus 1961 versus 2463
				IL-8	239030 versus 13629 versus 7
				IL-9	345 versus 42 versus 9
				IP-10	458 versus 513 versus 511
				MCP-1	63 versus 3201 versus 1902
				PDGF-AA	6667 versus 5568 versus 41
				PDGF-AB/BB	17 versus 1884 versus 75
				VEGF	4265 versus 538 versus 42

[9]	Hu-AF SC—Ckit (+)	Monolayer—16 hours	15 × 10^4^—70%/from 5 × 10^5^ cells Conc. 12.5x	VEGF, IL-8, SDF-1	1 ng/170.000 cell (1 *μ*g DNA)
				IL-6, MCP-1	0.5 ng/170.000 cell
				TGF-b	0.2 ng/170.000 cell
				IFNg	—
				IP-10 /CXCL10	—
				IL-1a	—

[20]	(i) Hu-AF-MSC (ii) Hu-AF-MSC-HPL	Monolayer—24 hours	1.5 × 10^6^ 80%/ Conc. 25x	Proteome analysis **Hu-AF-MSC-HPL-CM**: Anti-inflammatory cytokine: IL-10, IL-27, IL-17E, IL-13, IL-12p70, and IL-1ra Liver protection: MCP- 1, IL-1b **Hu-AF-MSC-HPL-CM, AF-MSC-CM**: Anti-inflammatory cytokine: TGF*β*1 Tissue repair: serpin E1, SDF-1 Angiogenesis: VEGF, PDEGF, and endostatin/collagen XVIII Liver regeneration: UPA, thrombospondin 1 and 2, HEGF, FGF-7, EGF, and HGF Anti-apoptotic markers: TIMP-1, IGFBP	

[8]	(i) Hu-PB-MNC- EPC (ii) HUVEC	Monolayer—72 hours	NA	IL-8/CXCL8 Hypox versus norm	29090.7 ± 12279.4 pg/mL versus 2282.1 ± 406.3 pg/mL
				SDF-1/CXCL12	6059.9 ± 654.6 pg/mL versus 3179.9 ± 488.0 pg/mL
				HGF	539.5 ± 141.7 pg/mL versus 343.4 ± 74.8 pg/mL
				Angiogenin	144.6 ± 68.2 pg/mL versus 72.5 ± 15.8 pg/mL
				PDGF-BB	111.6 ± 27.02 pg/mL versus 19.9 ± 2.2 pg/mL
				VEGF-A	25.5 ± 4.8 pg/mL versus 11.4 ± 5.2 pg/mL

[10]	Hu-UCB SC (endothelial + MSC)	Monolayer—48 hours	80%/Conc. 50x	EGF	3,286 ± 419 pg/mL
				VEGF	2,463 ± 151 pg/mL
				G-CSF	3,615 ± 173 pg/mL
				GM-CSF	3,623 ± 345 pg/mL
				TGF-*β*1, PDGF, bFGF, and KGF	=HUVEC
	HUVEC	Monolayer—48 hours	80%/Conc. 50x	EGF	UCB-SC-4.8X
				VEGF	UCB-SC-42x
				G-CSF	UCB-SC-3.7x
				GM-CSF	UCB-SC-2.4x

[22]	Hu-BM-MSC	Monolayer—24 hours	2 × 10^6^/Conc. 25x	69 from 174 prot. tested (+) (concentration NA)	

[27]	Hu-BM-MSC	Monolayer—24 hours	4 × 10^6^/Conc. 25x	VEGF HGF	Normoxia: 230 pg/mL Hypoxia: 450 pg/mL Normoxia: 600 pg/mL Hypoxia: 750 pg/mL

[17]	Hu-BM-MNC-stro-3-MPC	NA	1 × 10^6^/Conc.	IL-6 = 2x C	118.04 ± 0.27 pg/mL
				MCP-1 = 2x C	521.89 ± 1.48 pg/mL
				VEGF = 2x C	33.95 ± 2.98 pg/mL

[25]	(i) Hu-BM-MSC (ii) Hu-BM-CD133 (iii) Hu-BM-p75	Monolayer—48 hours	1 × 10^6^ 90%/ Conc. 40x	Secretion/cell	P75 versus CD133 versus BMMSC
				IL6—norm	3.8 versus 0.8 versus 0.6 fg
				IL6—hypox	0.25 = 0.25 versus 0.1 fg
				PlGF—norm	0.045 versus 0.01 versus 0 fg
				PlGF—hypox	0.043 versus 0.025 versus 0.15 fg
				ADM—norm	0.1 versus 0.05 versus 0.2 fg
				ADM—hypox	5.8 versus 5.4 versus 11.5 fg
				VEGF—norm	1.5 versus 1.0 versus 1.35 fg
				VEGF—hypox	0.7 versus 0.9 versus 0.95 fg
				SDF-1—norm	1.35 versus 0.75 versus 0.15 fg
				SDF-1—hypox	0.4 versus 0.7 versus 1.0 fg
				HGF—norm	0.84 versus 0.7 versus 0.25 fg
				HGF—hypox	0.01 versus 0.25 versus 0.01 fg
				DKK-I—norm	4 versus 4 versus 4.5 fg
				DKK-1—hypox	6.8 versus 6.5 versus 10.5 fg

[28]	Rat-BM-MSC	Monolayer—48 hours	90% T75/Conc. 40x	23 from 90 prot. tested (+)	
				NGF	356 ± 117 pg/mL
				BDNF	208 ± 57 pg/mL
				IL-6	427± 168 pg/mL

[24]	Hu-AD-MSC	Spheroid—2 days	4.2 × 10^7^/Centr.	CM versus *α*MEM:hTGF-b1	14.33 ± 6.71 versus 2.49 ± 2.39 pg/mL
				hVEGF	1,015.17 ± 170.97 pg/mL versus ND
				hPDGF-AA	Both ND

[6]	Hu-AD-SC In *α*MEM—FBS	Spheroid—2 days	10^5^/Centr.	VEGF	14.4 ± 0.4 ng/mL
				FGF2	13.2 ± 2.2 ng/mL
				HGF	13.3 ± 2.3 ng/mL
				CXCL12	16.6 ± 2.9 ng/mL
	In CRM-hu allo			No diff >< *α*MEM-FBS	
	In CRM-serum (−)			GF <	
	In *α*MEM—FBS	Monolayer—2 days	10^5^/Centr.	GF<<<	
	In CRM-hu allo				GF<<<

[5]	Hu-AD-SC	Monolayer— 72 hours	4 × 10^5^/Conc.	Spot density array hypox versus nomoxia	
				GCSF	14.07 ± 3.84 versus 10.13 ± 4.21
				GM-CSF	13.53 ± 1.26 versus 10.21 ± 1.44
				IGFBP-1	9.48 ± 0.44 versus 5.56 ± 0.44
				IGFBP-2	8.91 ± 0.02 versus 6.73 ± 0.31
				IGF-II	10.62 ± 0.85 versus 4.61 ± 0.93
				M-CSF	14.06 ± 0.13 versus 7.46 ± 1.69
				M-CSF R	9.09 ± 0.20 versus 3.31 ± 1.75
				PDGF R*β*	17.67 ± 1.32 versus 11.47 ± 1.40
				PDGF-AA	16.63 ± 1.33 versus 12.14 ± 2.12
				VEGF	13.47 ± 1.26 versus 5.59 ± 1.22
				EGF	11.06 ± 2.45 versus 34.14 ± 6.75

[36]	(i) AD-SC (ii) Hu dermal fibroblast	NA	NA	AD-SC	
				VEGF	810.65 ± 56.92 pg/*μ*g DNA
				IGF-I	328.33 ± 22.7 pg/*μ*g DNA
				Hu dermal fibroblast	
				VEGF	28.4 ± 2.25 pg/*μ*g DNA
				IGFI	Undetectable

[33]	Hu-MSC (Lonza)	Monolayer—48 hours	70%/(—)	IGF-1	1515.6 ± 211.8 pg/mL
				VEGF	465.8 ± 108.8 pg/mL
				TGF-b1	339.8 ± 14.4 pg/mL
				HGF	20.3 ± 7.9 pg/mL,
				FGF-2, PDGFBB, BMP-2, and SDF-1 (—)	

CRM: clinically relevant med, hu allo: human allogenic serum, MP: microparticle, ND: not detected, SDF-1: stromal derived factor-1, PlGF: placental GF, bFGF: basic FGF, HGF: hepatocyte GF, PAC: peripheral blood angiogenic cells (from PB MN cells-floating), cPAC: healthy control PAC, ESC-EC: ESC derived endothelial cell, MCP-1: monocyte chemotactic protein-1, PDEGF: platelet derived endothelial cell GF, UPA: urokinase plasminogen activator, HEGF: heparin binding epidermal GF, TIMP-1: tissue inhibitor of metalloproteinase-1, IGFBP: insulin-like GF binding protein, IP-10: interferon inducible protein-1, ADM: adrenomedullin, DKK-1: Dickkopf-1, norm: normoxic, hypox = hypoxic, fg = fentogram.

**Table 4:** Cell number to produce CM per application, volume, and mode of delivery of various cell sources for various conditions and the outcome.

Reference number	Condition/disease	Species	Cell source of CM	Culture medium/culture type—condition	Cell number/application	Volume and mode of delivery	Outcome
[6]	Hind limb ischemia—direct	Female athymic mice—20–25 gr	Hu-AD-SC	*α*MEM—FBS 10%/monolayer—hypox 1%	12.000	40 *μ*L—IM—7x	Good result
				CRM—Hu allo10%/spheroid—hypox 1%	48.000		Better result
				*α*MEM—FBS 10%/spheroid—hypox 1%			Better result

[9]	Hind limb ischemia—10 days	Male NOD-SCID mice—10–12 weeks	Hu-AF-SC–Ckit (+)	*α*MEM—(−)/monolayer—normoxia	500.000	80 *μ*L—IM—4x	Good result

[11]	Full thickness wound—5 mm direct	Diabetic-immunodef. mice—17–23 g	Hu-UCB-CD34-EPC	M199 basal medium (−)/monolayer—normoxia	1 × 10^6^	100 *μ*L—intradermal injection	Good result

[14]	Wound 30–50 mm^2^; 120–140 mm^2^—48 hours	Male NOD-SCID mice—4-5weeks	Hu-BM-MSC	*α*MEM—10% FBS/monolayer—normoxia	1 × 10^8^	100 *μ*L—SC—periphery wound	Good result

[17]	MCI 48 hours	Nude-athymic rat—6–8 weeks	Hu-BM-MNC-stro-3-MPC	*α*MEM—(−)/monolayer—normoxia	1 × 10^6^	250 *μ*L Intramyocardial	Good result

[20]	CCl4 injured acute hepatic failure—24 hours	SCID-NOD mice—6–8 weeks	Hu-AF-MSC	DMEM—0.5% FBS/monolayer—normoxia	1.5 × 10^6^	200 *μ*L—intrahepatic (left liver lobe)	Good result
			Hu-AF-MSC- HPL				Better result

[21]	Fulminant hepatic failure—24 hours	Male SD rat—250–300 g	Hu-MSC	DMEM—0.05% bovine serum albumin/monolayer—normoxia	1.5 × 10^6^	900 *μ*L penile vein	Good result Increased survival
[22]		Male SD rat—280–370 g	Hu-BM-MSC	NA—0.05% BSA/monolayer—normoxia	2 × 10^6^	900 *μ*L CM Penile vein	Good result Increased survival

[23]	Focal cerebral ischemia—72 hours	Male SD rat—350–400 g	Hu-EDT-SC	DMEM (−)/monolayer—normoxia	400.000	10x10 *µ*L—intranasal (left-right) Every day D3-D15	Good result
			BM-MSC (Lonza)				Good result

[24]	Ischemic Stroke—8 days	Male SD mouse—8 weeks	Hu-AD-MSC	*α*MEM—(−)/spheroid—hypoxia 1%	50.400	Infusion 0.5 *µ*L/hour-7 days—lateral ventricle	Good result

SCID: severe combined immunodeficiency, NOD: nonobese diabetic, SD: Sprague-Dawley, Hu: human, AD: adipose tissue, SC: stem cell, AF: amniotic fluid, UCB: umbilical cord blood, EPC: endothelial progenitor cell, BM: bone marrow, MSC: mesenchymal SC, MNC: mononuclear cell, MPC: mesenchymal progenitor cell, HPL: hepatic progenitor-like cell, and EDT: exfoliated deciduous tooth.
